# Identification of candidate genes involved in the sugar metabolism and accumulation during pear fruit post-harvest ripening of ‘Red Clapp’s Favorite’ (*Pyrus communis* L.) by transcriptome analysis

**DOI:** 10.1186/s41065-017-0046-0

**Published:** 2017-09-21

**Authors:** Long Wang, Yun Chen, Suke Wang, Huabai Xue, Yanli Su, Jian Yang, Xiugen Li

**Affiliations:** Zhengzhou Fruit Research Institute, Chinese Academy of Agricultural Sciences, Weilai Road South End, Zhengzhou, Henan 450009 China

**Keywords:** Pear (*Pyrus communis* L.), Quantitative real-time PCR (qPCR), RNA-Seq, Sugar determination

## Abstract

**Background:**

Pear (*Pyrus* spp.) is a popular fruit that is commercially cultivated in most temperate regions. In fruits, sugar metabolism and accumulation are important factors for fruit organoleptic quality. Post-harvest ripening is a special feature of ‘Red Clapp’s Favorite’.

**Results:**

In this study, transcriptome sequencing based on the Illumina platform generated 23.8 - 35.8 million unigenes of nine cDNA libraries constructed using RNAs from the ‘Red Clapp’s Favorite’ pear variety with different treatments, in which 2629 new genes were discovered, and 2121 of them were annotated. A total of 2146 DEGs, 3650 DEGs, 1830 DEGs from each comparison were assembled. Moreover, the gene expression patterns of 8 unigenes related to sugar metabolism revealed by qPCR. The main constituents of soluble sugars were fructose and glucose after pear fruit post-harvest ripening, and five unigenes involved in sugar metabolism were discovered.

**Conclusions:**

Our study not only provides a large-scale assessment of transcriptome resources of ‘Red Clapp’s Favorite’ but also lays the foundation for further research into genes correlated with sugar metabolism.

**Electronic supplementary material:**

The online version of this article (10.1186/s41065-017-0046-0) contains supplementary material, which is available to authorized users.

## Background

Pear (*Pyrus* spp.), one of the most important and oldest temperate fruit tree species (belonging to the subfamily Pomoideae in the family Rosaceae), has been grown in temperate regions since antiquity in both Europe and China [1]. A large number of pear cultivars are functional diploids (2n = 34). The primary edible pear species are the Japanese pear (*Pyrus pyrifolia* Nakai), the European pear (*P. communis* L.) and Chinese pears (*P. bretschneideri* Rehd. and *P. ussuriensis* Maxim.), which are grown for commercial fruit production. The Japanese pear and Chinese pears are grown in East Asia, while the European pear is cultivated in Europe and other temperate regions of the Southern Hemisphere [2].

Improving fruit quality has become an important direction of fruit tree cultivation. In fruits, sugar metabolism and accumulation are important factors for fruit organoleptic quality. Sugar, the primary product of photosynthesis and a substrate of respiration, is required for carbon skeleton construction and energy supply in plants [3]. Sugars are known to play key roles in both plant metabolic and defense responses as signaling molecules [4–8]. Moreover, soluble sugars (sucrose, glucose and fructose) are important components of fruit taste, directly influencing consumer preferences for fresh fruit [9]. Glucose and fructose take part in cell division, and sucrose is actively involved in differentiation and maturation [10]. It has been reported that the levels and ratios of these sugars differ in various tree species and rely on the major catalytic enzymes in sugar metabolism [11]. Soluble acid invertase (INV) converts sucrose into fructose and glucose [12]. In pear and many other woody Rosaceae plants, photosynthetic products, primarily in the form of sorbitol, are produced by leaves and transported to the fruit and other organs, which leads to the invertase-catalyzed hydrolysis of sorbitol to glucose and fructose [3]. It is well known that environmental factors (such as temperature and light) have a certain impact on sugar metabolism in post-harvest fruit [7]. For example, Wang [13] showed that peach fruit stored at 5 °C produced lower levels of sucrose and higher levels of glucose and fructose than fruit stored at 0 °C. It is well documented that the accumulation of soluble sugars could be improved by modifying the enzymatic activity of sucrose metabolism of post-harvest lemon fruit after exposure to UV-B [14]. Transcriptome sequencing has become a powerful tool to profile transcriptomes due to its reproducibility, sensitivity, high throughput, low cost and accuracy [15]. Transcriptome sequencing is an effective technique for the acquisition of sequences for new genes and provides opportunities to study specific cellular pathways and gene expression patterns [16–18]. In this work, expression data regarding differentially expressed genes were analyzed, and the respective putative functions of the sequences were identified through the described screening process. Our study aimed to provide important information for further functional studies of novel genes of ‘Red Clapp’s Favorite’ related to sugar metabolism and accumulation using RNA-Seq technology.

## Methods

### Plant materials and treatments

The plant materials of ‘Red Clapp’s Favorite’ (*Pyrus communis* L.) used in this study were obtained from Zhengzhou Fruit Research Institute, Chinese Academy of Agricultural Sciences in Henan Province, China. Group 1 (T04, T07 and T10) of the pear pulps was collected at maturity. Group 2 (T05, T08 and T11) of the pear pulps was subjected to low temperature (5 °C) for 10 days after picking. Group 3 (T06, T09 and T12) was treated at normal temperature (25 °C) for 3 days after treatment at low temperature (5 °C, 10 days), the fruit went soft. All of the treatments were performed using three replicates with three fruits for each replicate. All collected samples were immediately frozen in liquid nitrogen and stored at −80 °C until RNA extraction.

### RNA extraction and cDNA library construction

The extraction of RNA, construction of cDNA libraries and the transcriptome sequencing assay were performed by Biomarker Biotechnology Corporation (Beijing, China). RNA degradation and contamination were checked on 1% agarose gels. RNA purity and concentration were measured using the NanoPhotometer spectrophotometer (IMPLEN, CA, USA) and Qubit RNA Assay Kit in Qubit 2.0 Fluorometer (Life Technologies, CA, USA). RNA integrity was assessed using the RNA Nano 6000 Assay Kit of the Agilent Bioanalyzer 2100 system (Agilent Technologies, CA, USA). Sequencing libraries were generated using the NEBNext UltraTM RNA Library Prep Kit for Illumina (NEB, USA) according to the manufacturer’s recommendations. The library quality was monitored on the Agilent Bioanalyzer 2100 system. The first-strand cDNA synthesis for qPCR was obtained by the PrimeScript™ RT reagent Kit with gDNA Eraser (Perfect Real Time) (TaKaRa, Japan).

### Data analysis and functional annotation

Clean data (clean reads) were acquired by trimming reads containing adapters and those containing poly-N and low-quality reads from raw data. Concurrently, the Q20, Q30, GC content and sequence duplication levels of the clean data were calculated. All the analyses were based on clean data with high quality. Gene function was annotated based on the following downstream databases: Nr (NCBI non-redundant protein sequences); Nt (NCBI non-redundant nucleotide sequences); Pfam (Protein family); KOG/COG (Clusters of Orthologous Groups of proteins); Swiss-Prot (a manually annotated and reviewed protein sequence database); KO (KEGG Ortholog database); and GO (Gene Ontology). GO and KEGG were also used to classify unigene functions. In addition, the complex biological behaviors of unigenes and pathway annotation for unigenes were further studied by KEGG annotation. Quantification of gene expression levels was estimated by fragments per kilobase of transcript per million fragments mapped (FPKM) using the following formula:

$$ \mathrm{FPKM}=\frac{\mathrm{cDNA}\  \mathrm{Fragments}}{\mathrm{Mapped}\  \mathrm{Fragments}\left(\mathrm{Millions}\right)\times \mathrm{Transcript}\  \mathrm{Length}\left(\mathrm{kb}\right)} $$

In this formula, cDNA Fragments represents the number of fragments that aligned to a specific transcript. Mapped Fragments (Millions) represents the total number of fragments that aligned to all transcripts. Transcript Length (kb) represents the length of the transcript.

### Identification of differentially expressed genes (DEGs)

Differentially expressed genes (DEGs) between two groups were identified using the DESeq R package. DESeq provides statistical routines for determining differential expression in digital gene expression data using a negative binomial distribution model. The resulting *P* values were adjusted using Benjamini and Hochberg’s approach for controlling the false discovery rate. Genes with an adjusted *P*-value <0.05 found by DESeq were considered differentially expressed.

### Real-time quantitative PCR analysis and sugar content determination

Real-time quantitative PCR (qPCR) was performed following the manufacturer’s protocol of the SYBR Green I Master (ROX) (Roche, USA) using the LightCycler480 real-time PCR system (Roche, USA). The qPCR procedure was as follows: 50 °C, 2 min; 95 °C, 10 min; and 40 cycles of 94 °C, 15 s and 60 °C, 60 s. The qPCR results were analyzed by the 2^-△△Ct^ method [19]. Tubulin (AB239681) was used as the reference gene. The primers for selected DEGs and tubulin are shown in Table 1 and were designed using Beacon Designer7 and synthesized by GENEWIZ (Suzhou, China).

**Table 1:** The primers for selected DEGs and Tubulin

Gene	Forward primer (5′-3′)	Reverse primer (5′-3′)
PCP011895	CTTCAAGGATTGCCACAT	TGTATCGTATTATTCAAGTAGAGG
PCP005049	ACATCACAATAGGTTCTG	TCTCAACTTCACATTCTG
PCP013141	AACCACCTCTTCTGATGA	ACCACAAGTAGTCTGTAGTA
PCP008001	GGTCATATTCCTCTTGTCATTAG	TTGTTCTGGCACTGTCTT
PCP030959	TCGGTCATATTCCTCTTGTC	TTGTTCTGGCACTGTCTT
PCP006674	GCTTGGTCTTGAATATGA	CGGCAGAATCTTAATGTA
PCP005278	TAGCCAGAGCAATAAGGA	GGAGTTCGTTCAAGTGTT
PCP012345	GTAGCAGTGGATTCATAGC	GCATCAGTAGCATTGGTT
newGene_4807	CAAGAAGCAGCAAGAGAA	CACTAGGAATCAAGGCATC
PCP005062	GCTTCTGTTGTTATCTCCTCTA	TTCCACCACTGCTGATTG
Tubulin	TGGGCTTTGCTCCTCTTAC	CCTTCGTGCTCATCTTACC

A 100 mg sample of each pear was weighed and extracted for LC-ESI-MS/MS of sugar content determination. Methanol, acetonitrile and ethanol were purchased from Merck Company (Germany). Standards were purchased from Sigma-Aldrich, were dissolved in methanol and preserved at −20 °C for LC-MS analysis.

## Results

### Sequencing

Nine cDNA libraries, T04, T07, T10, T05, T08, T11, T06, T09 and T12, were sequenced on the Illumina HiSeq 2500 platform, which generated a total of 23.8 - 35.8 million clean reads of each library after data filtering and stringent quality investigation (Table 2). The GC content of each clean data was below 50%, with a quality score (Q30) percentage above 94% (Table 2), demonstrating that the reliability and quality of the sequencing data were adequate for further analysis. The ratio of mapped reads ranged from 74.37% to 76.99% (Table 2). Based on the mapped results, 2629 new genes were discovered, and 2121 of them were annotated (Table 3). In addition, 27 DEGs, 44 DEGs, 171 DEGs and 197 DEGs fall into “up-up”, “down-down”, “down-up” and “down-up” pattern along with three treatments (G1 vs. G2 vs. G3), respectively (Table 4).

**Table 2:** Summary statistics of Illumina sequencing for ‘Red Clapp’s Favorite’

Samples	Clean reads	GC Content	% ≥ Q30	Mapped reads ratio
T04	23,800,724	47.34%	94.28%	76.02%
T05	35,882,930	47.03%	94.78%	75.87%
T06	29,358,478	47.44%	94.75%	74.40%
T07	31,642,033	47.49%	94.74%	76.99%
T08	29,876,598	47.55%	94.15%	75.30%
T09	26,183,442	47.21%	94.69%	74.37%
T10	33,015,521	46.83%	94.83%	76.61%
T11	32,051,417	47.10%	95.12%	76.99%
T12	30,183,239	47.33%	94.54%	75.75%

Clean reads represent total pair-end reads of clean data; Mapped reads ratio represents the percent of clean data mapped to the pear reference genome (http://www.rosaceae.org/species/pyrus/pyrus_communis/genome_v1.0)

**Table 3:** Statistical table for the annotation of new genes

Annotated databases	New gene number
COG	285
GO	772
KEGG	584
eggNOG	1636
nr	2114
All	2121

**Table 4:** Number of DEGs fall into four patterns

Patterns	Number of DEGs
Up-up	27
Down-down	44
Down-up	171
Up-down	197

### Analysis of DEGs

**Fig. 1:**
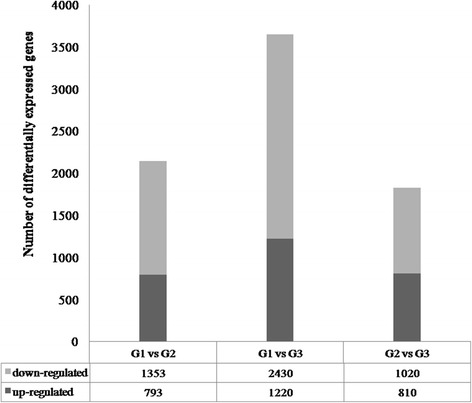
Differentially expressed genes in each comparison

**Fig. 2:**
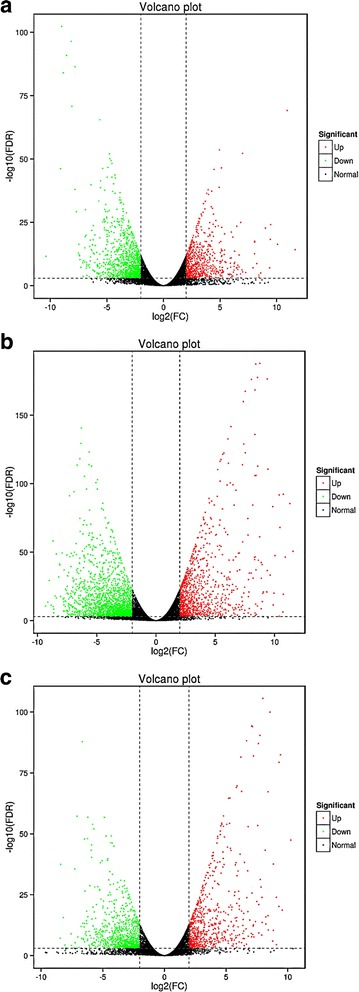
Volcano plot of differentially expressed genes in each comparison. The *x*-axis indicates log2 FC (fold changes) between the two samples and the *y*-axis indicates the -log10 FDR (false discovery rate) of gene expression variation. The up-regulated genes are shown as red dots, the down-regulated genes are shown as green dots and the normal genes are shown as black dots. Note: **a** Volcano plot of DEGs in the G1 vs. G2 comparison; **b** volcano plot of DEGs in the G1 vs. G3 comparison; **c** volcano plot of DEGs in the G2 vs. G3 comparison

To obtain DEGs among three biological replicates, the samples were identified via two-two comparisons: Group 1 vs. Group 2 (G1 vs. G2), Group 1 vs. Group 3 (G1 vs. G3) and Group 2 vs. Group 3 (G2 vs. G3). In the G1 vs. G2 comparison, 2146 genes confirmed significantly different expression, including 793 DEGs that were up-regulated and 1353 DEGs that were down-regulated (Fig. 1). Figure 1 shows that there were 1220 up-regulated and 2430 down-regulated DEGs in the G1 vs. G3 comparison. Among the 1830 DEGs in the G2 vs. G3 comparison, 810 DEGs were up-regulated and 1020 DEGs were down-regulated. After screening all differentially expressed genes, we constructed a volcano plot to observe the DEGs more clearly (Fig. 2).

### GO classification

**Fig. 3:**
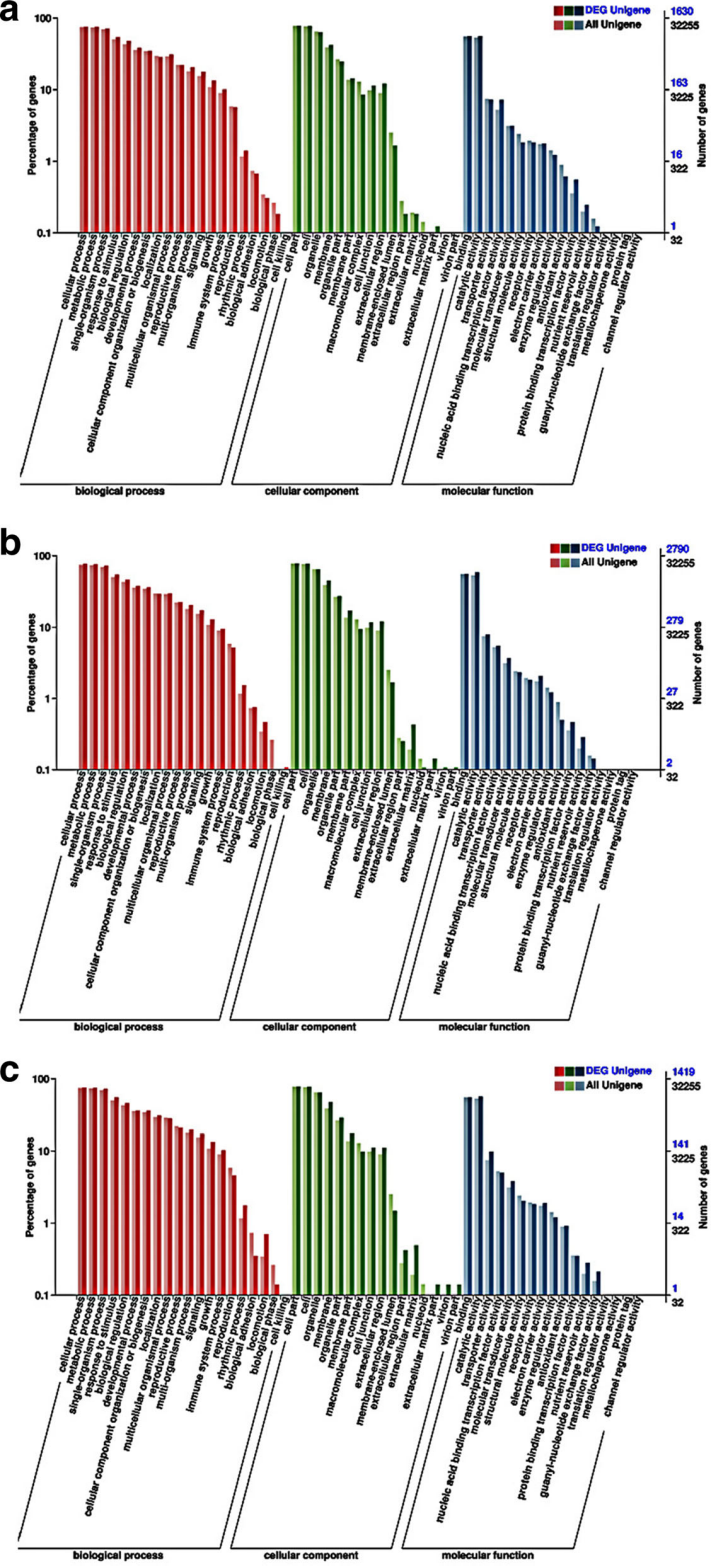
GO classification of each comparison. The right side of the *y*-axis indicates the number of genes, and the left side of the *y*-axis indicates the percentage of genes. Note: **a** Sequencing from the G1 vs. G2 comparison; **b** sequencing from the G1 vs. G3 comparison; **c** sequencing from the G2 vs. G3 comparison

GO, an international standardized gene functional classification system, defines both the concepts/classes used to describe gene function and the relationships between these concepts and can adjust for gene length bias in DEGs. The total number of unigenes among all three comparisons was 32,255, including 1630 DEGs in the G1 vs. G2 comparison, 2790 DEGs in the G1 vs. G3 comparison and 1419 DEGs in the G2 vs. G3 comparison, which were assigned to three main GO categories, which included biological process, molecular function and cellular component (Fig. 3). All of them were assigned to 53 functional groups using GO assignments (Fig. 3). Figure 3a shows that the DEGs in the G1 vs. G2 comparison were significantly enriched in GO terms such as “signaling”, “growth” and “rhythmic process” in the “biological process” category; “extracellular region” in the “cellular component” category; and “nucleic acid binding transcription factor activity” and “protein binding transcription factor activity” in the “molecular function” category. Figure 3b shows that DEGs of “rhythmic process” and “locomotion” in the “biological process” category, “extracellular matrix” and “extracellular matrix part” in the “cellular component” category, and “protein binding transcription factor activity” and “nutrient reservoir activity” in the “molecular function” category were found to be significantly enriched in the GO terms in the G1 vs. G3 comparison. Figure 3c shows that DEGs in the G2 vs. G3 comparison were significantly enriched in GO terms such as “rhythmic process” and “locomotion” in the “biological process” category; “extracellular region part”, “extracellular matrix”, “extracellular matrix part”, “virion” and “virion part” in the “cellular component” category; and “transporter activity”, “nutrient reservoir activity” and “guanyl-nucleotide exchange factor activity” in the “molecular function” category.

### Unigenes for sugar metabolism analysis and qPCR validation

**Fig. 4:**
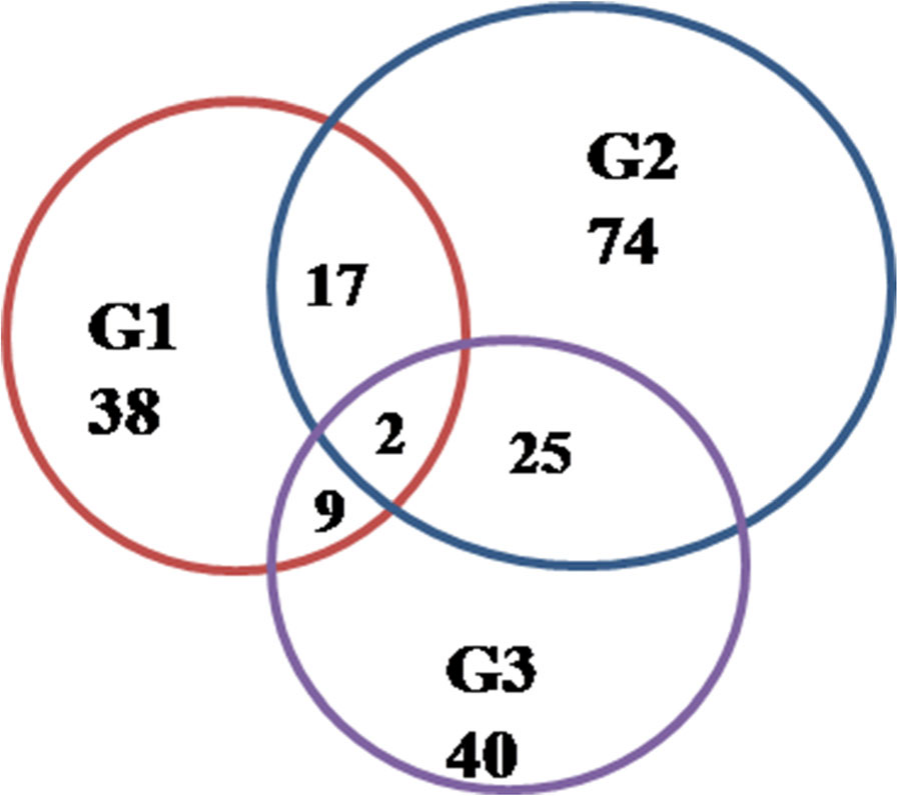
Numbers of DEGs related to sugar metabolism in the three groups

Among the 2146 unigenes in the G1 vs. G2 comparison, 771 unigenes could be annotated to the KEGG, including 38 annotated unigenes related to sugar metabolism (fructose and mannose metabolism, galactose metabolism, and starch and sucrose metabolism) (Fig. 4). Among the 3650 and 1830 unigenes in the G1 vs. G3 comparison and G2 vs. G3 comparison, 1412 and 687 unigenes could be annotated to the KEGG, including 74 and 40 annotated unigenes related to sugar metabolism (fructose and mannose metabolism, galactose metabolism and starch and sucrose metabolism), respectively (Fig. 4). Figure 4 shows that half of the unigenes between G1 and G2 were similarly expressed, and similar results were found between G2 and G3. Two unigenes were expressed in all three groups (Fig. 4).

**Fig. 5:**
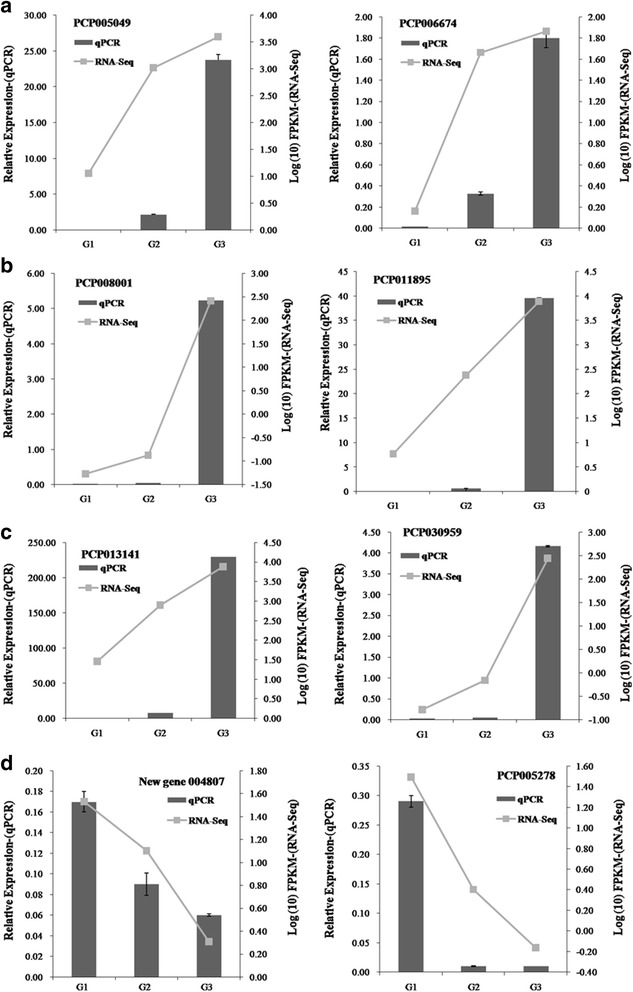
**a** Expression of PCP005049 gene and PCP006674 gene as determined by RNA-Seq and qRT-PCR; **b** Expression of PCP008001 gene and PCP011895 gene as determined by RNA-Seq and qRT-PCR; **c** Expression of PCP013141 gene and PCP030959 gene as determined by RNA-Seq and qRT-PCR; **d** Expression of new gene 004807 and PCP005278 gene as determined by RNA-Seq and qRT-PCR. The details of above genes were showed in Table S1. Tubulin (AB239681) was used as the reference gene. The pear reference genome was on <http://www.rosaceae.org/species/pyrus/pyrus_communis/genome_v1.0>

To validate the reliability and accuracy of the RNA-Seq results, 8 candidate genes associated with sugar metabolism (galactose metabolism and starch and sucrose metabolism) were randomly selected for RT-qPCR assays, including 6 up-regulated unigenes (PCP005049, PCP006674, PCP008001, PCP011895, PCP013141 and PCP030959) (Fig. 5a–c) and 2 down-regulated unigenes (PCP005278 and a novel gene, 004807) (Fig. 5d). The details of these unigenes are shown in Additional file 1: Table S1, the pathways which they involved in are shown in Additional file 2: Figure S1 and Additional file 3: Figure S2. The results from the qPCR analysis demonstrated that nearly all of these genes showed similar expression trends to those of RNA-Seq (Fig. 5a–d).

### Analysis of sugar content and related genes

**Fig. 6:**
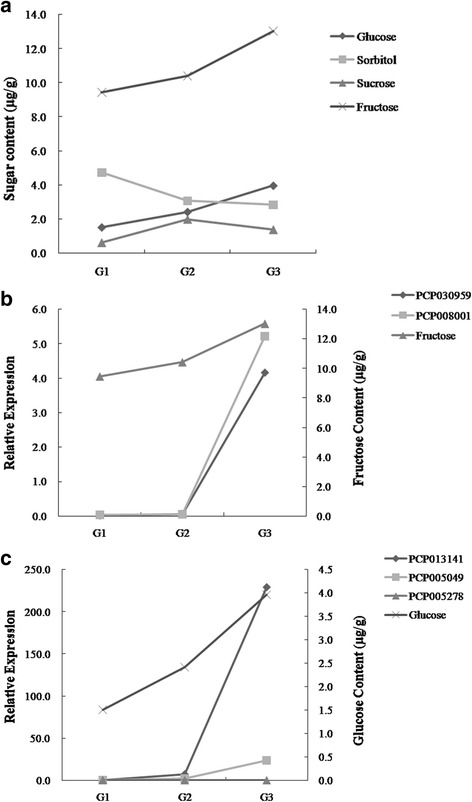
**a** Sugar (glucose, sorbitol, sucrose and fructose) content in ‘Red Clapp’ pears during different periods; **b** The correlation between fructose content and its related unigenes’ expression; **c** The correlation between glucose content and its related unigenes’ expression

As Fig. 6a shows, the main constituents of soluble sugars, which consisted of fructose and glucose, were increased gradually, while sorbitol was decreased with the pear fruit post-harvest ripening process. In addition, sucrose was increased first and then decreased. Fructose was the most abundant soluble sugar during the pear post-harvest ripening period (G1-G2-G3) in pear (Fig. 6a). Sorbitol was the second most abundant soluble sugar at fruit maturation (G1) (Fig. 6a).

Five of the abovementioned unigenes that were related to sugar metabolism were selected. The relative expression of two unigenes (PCP030959 and PCP008001) increased rapidly with the increase in fructose content (Fig. 6b). The unigene PCP013141 was significantly increased with the increase in glucose content (Fig. 6c). The unigene PCP005278 showed a small increase with the increase in glucose content (Fig. 6c).

## Discussion

RNA-Seq is a feasible and economical way to detect genes of interest in a short time, and its popularity among researchers continues to increase [20–22]. In modern times, transcript information, gene structure and functional annotation, alternative splicing, and DEGs can be obtained by RNA-Seq technology [23, 24]. This technology has been widely applied to model and non-model species, such as Arabidopsis (*Arabidopsis thaliana*) [25], rice (*Oryza sativa*) [26, 27], cotton (*Gossypium hirsutum*) [28], licorice (*Glycyrrhiza uralensis* Fisch) [29], *Scutellaria baicalensis* Georgi [30], and Chinese wolfberry (*Lycium chinense* Mill.) [31]. In this study, a total of 23.8 - 35.8 million clean reads were obtained from 9 cDNA libraries using this technology (Table 2) and were assembled into a total of 2146 DEGs, 3650 DEGs, and 1830 DEGs, respectively, from each comparison (Fig. 1).

Great taste is a prerequisite for consumer satisfaction [32]. Pear eating quality is influenced by climatic conditions, post-storage ripening and harvest time [33]. Physiological maturity of ‘Red Clapp’s Favorite’ is the stage of development when the fruit ripens adequately after harvest [34]. Hence, fruit ripening processes are very important because they influence the changes that appear during fruit storage, transport and shelf life and changes in aroma and color [35]. Sugar metabolism is an important part of the pear ripening process. In this research, we used RNA-Seq technology to study DEGs in the ripening process. These DEGs showed functional diversity. In the GO functional analysis, DEGs were involved in extracellular region, extracellular region part, extracellular matrix, extracellular matrix part, virion and virion part of the cellular component category (Fig. 3a–c). The DEGs were involved in molecular function categories such as nucleic acid binding transcription factor activity, protein binding transcription factor activity, protein binding transcription factor activity, nutrient reservoir activity, transporter activity, nutrient reservoir activity and guanyl-nucleotide exchange factor activity (Fig. 3a–c). The DEGs were also involved in the biological process category, including signaling, growth, rhythmic process and locomotion (Fig. 3a–c). All of these results show that sugar metabolism is a complex physiological and biochemical process.

We analyzed the eight differentially expressed unigenes related to sugar metabolism with qPCR, and the results showed similar expression trends to those of RNA-Seq (Fig. 5a–d). Only the multiple of up-regulated or down regulated was different, which validated that the RNA-Seq analysis was generally more accurate and robust. Soluble sugars, including fructose, sucrose, glucose and sorbitol, are an important factor in determining fruit quality and flavor. Fructose showed the highest sweetness, followed by sucrose; the sweetness of glucose and sorbitol was the lowest. Chen et al. showed that fructose was the dominant sugar in eight pear varieties, followed by glucose and sucrose [36]. In this study, fructose was the main sugar after pear post-harvest ripening, followed by glucose, sucrose and sorbitol. This result might indicate that fructose is the key factor in the pear eating quality of ‘Red Clapp’ (Fig. 6a). Interestingly, we discovered five unigenes that might be involved in sugar metabolism (Fig. 6b, c). In future studies, we plan to clone the specific genes related to sugar metabolism and verify their functions.

## Conclusions

In this study, transcriptome sequencing was performed on the Illumina platform, generating 23.8 - 35.8 million unigenes of nine cDNA libraries constructed using RNAs from the ‘Red Clapp’s Favorite’ pear variety with different treatments. A number of DEGs and novel genes were obtained from each group and were assembled. Moreover, the gene expression patterns of 8 unigenes related to sugar metabolism revealed by qPCR confirmed the RNA-Seq data. The main constituents of soluble sugars were fructose and glucose after pear fruit post-harvest ripening, and five unigenes involved in sugar metabolism were discovered. This study lays the foundation for further research into genes correlated with sugar metabolism.

## Additional files

Additional file 1: Table S1. KEGG annotation details of unigenes (for qPCR). (DOCX 12 kb)

Additional file 2: Figure S1. The pathway of starch and sucrose metabolism. Note: PCP008001 and PCP030959 were involved in the process of 3.2.1.26 (Marked in red); PCP011895 was involved in the process of 3.1.1.11 (Marked in blue); PCP006674 was involved in the process of 2.4.1.15 and 3.1.3.12 (Marked in blue); a novel gene 004807 was involved in the process of 2.7.7.27 (Marked in green). (JPEG 396 kb)

Additional file 3: Figure S2. The pathway of galactose metabolism. Note: PCP005049 and PCP013141 were involved in the process of 3.2.1.23 (Marked in red); PCP005278 was involved in the process of 3.2.1.108 (Marked in green). (JPEG 324 kb)
