# Supplementary material for: Identification of candidate genes involved in the sugar metabolism and accumulation during pear fruit post-harvest ripening of ‘Red Clapp’s Favorite’ (Pyrus communis L.) by transcriptome analysis
Source: Hereditas. 2017 Sep 21;155:11. doi: 10.1186/s41065-017-0046-0 (PMC5609059; doi:10.1186/s41065-017-0046-0)
Supplement: Supplementary file 1 — KEGG annotation details of unigenes (for qPCR). (DOCX 12 kb) [file 41065_2017_46_MOESM1_ESM.docx]

**Table S1 The KEGG annotation details of unigenes (for qPCR)**

| Unigene ID | KEGG annotation [international enzyme name] |
| --- | --- |
| PCP005049 | beta-galactosidase [EC:3.2.1.23] |
| PCP006674 | alpha-trehalose-phosphate synthase |
| PCP008001 | beta-fructofuranosidase [EC:3.2.1.26] |
| PCP011895 | pectinesterase [EC:3.1.1.11] |
| PCP013141 | beta-galactosidase [EC:3.2.1.23] |
| PCP030959 | beta-fructofuranosidase [EC:3.2.1.26] |
| New Gene 004807 | glucose-1-phosphate adenylyltransferase [EC:2.7.7.27] |
| PCP005278 | beta-glucosidase [EC:3.2.1.21] |
