# Supplementary figures and images for: Identification of candidate genes involved in the sugar metabolism and accumulation during pear fruit post-harvest ripening of ‘Red Clapp’s Favorite’ (Pyrus communis L.) by transcriptome analysis
Source: Hereditas. 2017 Sep 21;155:11. doi: 10.1186/s41065-017-0046-0 (PMC5609059; doi:10.1186/s41065-017-0046-0)

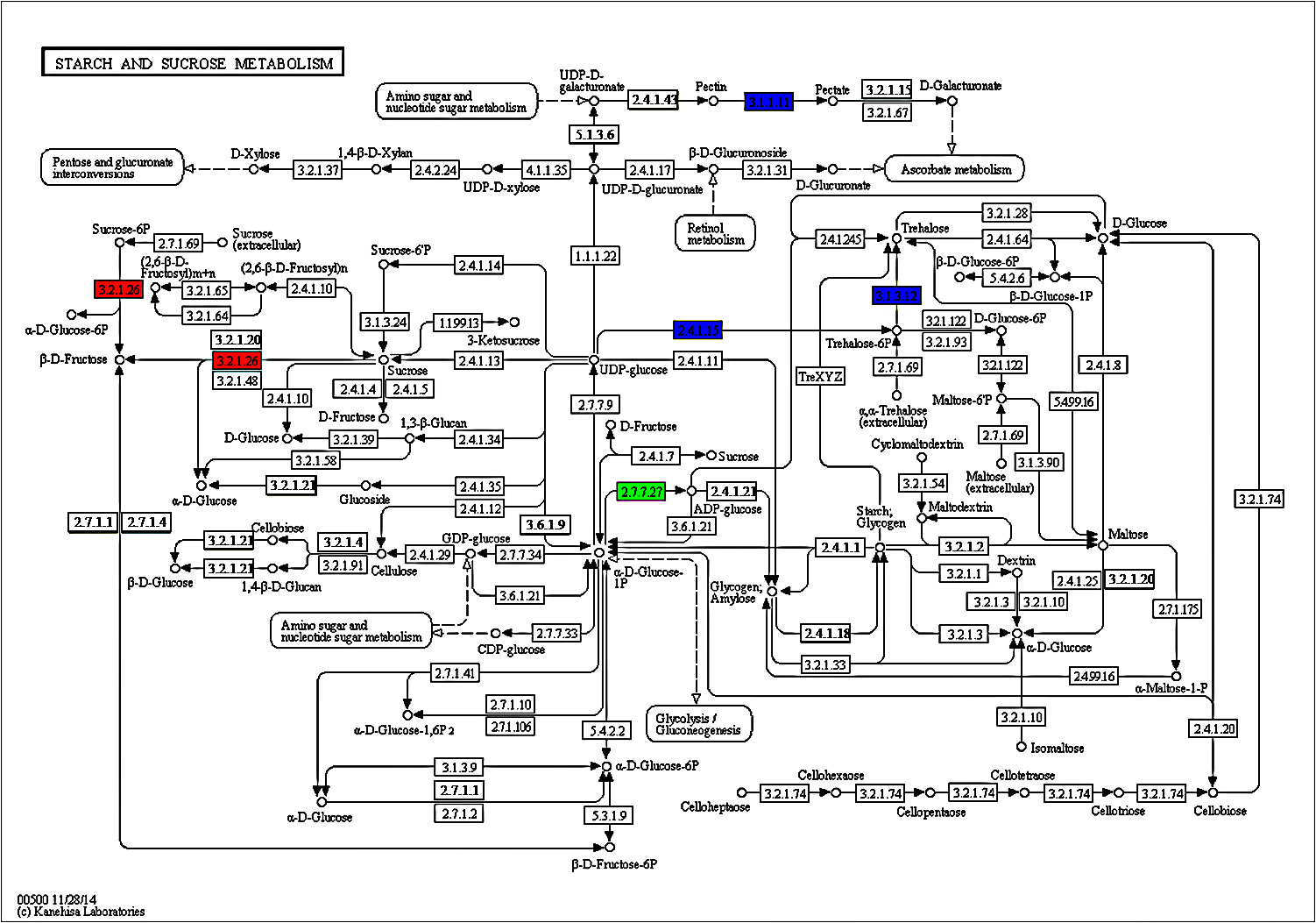

Supplement: Supplementary file 2 — The pathway of starch and sucrose metabolism. Note: PCP008001 and PCP030959 were involved in the process of 3.2.1.26 (Marked in red); PCP011895 was involved in the process of 3.1.1.11 (Marked in blue); PCP006674 was involved in the process of 2.4.1.15 and 3.1.3.12 (Marked in blue); a novel gene 004807 was involved in the process of 2.7.7.27 (Marked in green). (JPEG 396 kb) [file 41065_2017_46_MOESM2_ESM.jpg]

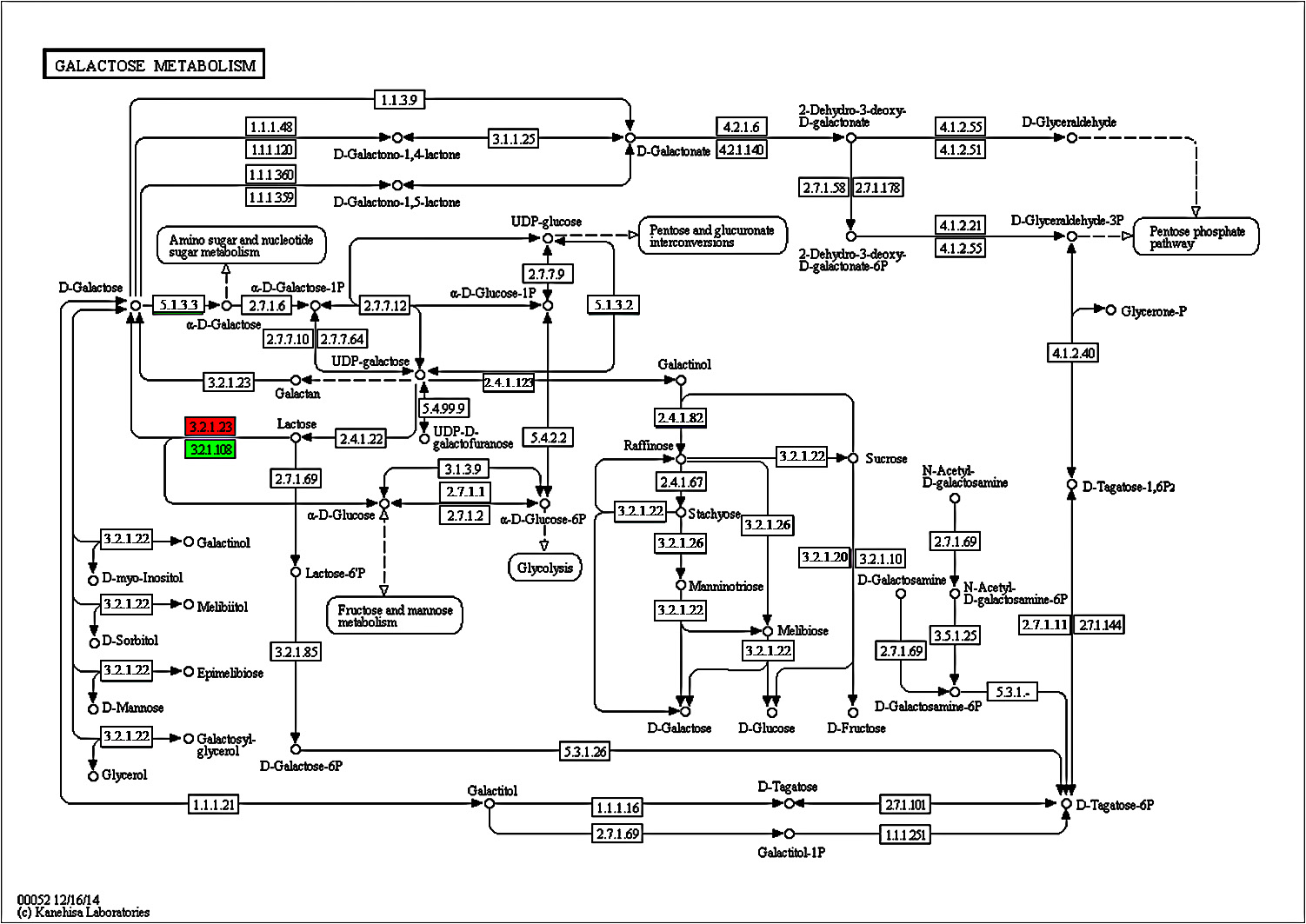

Supplement: Supplementary file 3 — The pathway of galactose metabolism. Note: PCP005049 and PCP013141 were involved in the process of 3.2.1.23 (Marked in red); PCP005278 was involved in the process of 3.2.1.108 (Marked in green). (JPEG 324 kb) [file 41065_2017_46_MOESM3_ESM.jpg]
